# Health insurance for people with citizenship problems in Thailand: a case study of policy implementation within a complex health system

**DOI:** 10.1186/1472-6963-14-S2-P121

**Published:** 2014-07-07

**Authors:** Rapeepong Suphanchaimat, Weerasak Putthasri, Phusit Prakongsai, Anne Mills

**Affiliations:** 1 London School of Hygiene and Tropical Medicine, London, UK; 2 International Health Policy Programme, The Ministry of Public Health, Nonthaburi, Thailand

## Background

Health care provision for non-citizens (illegal migrants, stateless people, etc) is a common problem across the world. Since 2002, Thailand has achieved universal health coverage through the introduction of the Universal Coverage Scheme (UCS), covering almost all residents. However, people with citizenship problems, so-called ‘stateless people’, were left uninsured. Consequently, the ‘Health Insurance for People with Citizenship Problems’ (HIS-PCP) policy was adopted in 2010. This study sought to examine operational constraints facing the implementation of the policy, through the views of ground-level providers.

## Materials and methods

A qualitative, case-study approach was devised. Individual in-depth interviews and group interviews with 33 key informants (3 ministerial officers, 4 hospital directors, 2 provincial chief medical officers, and 24 ‘street-level bureaucrats’) were conducted in Tak and Ranong provinces. Interview data were triangulated with document reviews and observation. Framework analysis was applied for data analysis. Interview data and relevant codes were mapped and interpreted against the six health-system building blocks and the issues of legality and patient characteristics.

## Results

The policy faced several operational problems from all health-system angles. Inadequate communication and unclear service guidelines contributed to the ineffectiveness in budget spent and services provision. The problems were linked with the regulation concerning patient referral, which contradicted the legal requirements imposed on, and the highly mobile behaviour of, the stateless people. Some providers adapted their practices to meet on-the-job difficulties, including establishing a mutual agreement between neighbouring hospitals to allow stateless patients to bypass the primary care gatekeeper, but this then created a sense of unfair treatment amongst UCS beneficiaries. These challenges were intertwined with official procrastination over nationality verification procedures and poor collaboration between ministries.

## Conclusion

The HIS-PCP encountered various constraints along its implementation. Inadequate communication and discordance between policy objectives and perceptions of healthcare staff were key explanations. Impractical legal instruments and distinctive behaviours/characteristics of stateless people made the problems more complex. Policy recommendations were suggested. In the short term, technical and human-resources capacities of the scheme’s governing body should be strengthened. Communications between the authorities within the Ministry of Public Health (MOPH) and collaboration with the Ministry of Interior, should be improved. Guidelines concerning budgeting and scope of service provision should be fine-tuned. In the long run, the nationality verification of stateless people should be expedited. The MOPH should develop clear and practical guidelines to assist health personnel to cope with citizenship problems of patients, which are beyond routine clinical services.

